# Development of spatial density maps based on geoprocessing web services: application to tuberculosis incidence in Barcelona, Spain

**DOI:** 10.1186/1476-072X-10-62

**Published:** 2011-11-29

**Authors:** Pau Dominkovics, Carlos Granell, Antoni Pérez-Navarro, Martí Casals, Àngels Orcau, Joan A Caylà

**Affiliations:** 1 Estudis d'Informàtica, Multimèdia i Telecomunicació, Universitat Oberta de Catalunya (UOC), Rambla del Poblenou, 156, 08018, Barcelona, Spain; 2 Institute of New Imaging Technologies, Universitat Jaume I de Castellón (UJI), Castellón, Spain; 3 Programa de Prevenció i Control de la Tuberculosi de Barcelona, Servei d'Epidemiologia, Agència de Salut Pública de Barcelona, CIBER de Epidemiología y Salud Pública (CIBERESP), Barcelona, Spain

## Abstract

**Background:**

Health professionals and authorities strive to cope with heterogeneous data, services, and statistical models to support decision making on public health. Sophisticated analysis and distributed processing capabilities over geocoded epidemiological data are seen as driving factors to speed up control and decision making in these health risk situations. In this context, recent Web technologies and standards-based web services deployed on geospatial information infrastructures have rapidly become an efficient way to access, share, process, and visualize geocoded health-related information.

**Methods:**

Data used on this study is based on Tuberculosis (TB) cases registered in Barcelona city during 2009. Residential addresses are geocoded and loaded into a spatial database that acts as a backend database. The web-based application architecture and geoprocessing web services are designed according to the Representational State Transfer (REST) principles. These web processing services produce spatial density maps against the backend database.

**Results:**

The results are focused on the use of the proposed web-based application to the analysis of TB cases in Barcelona. The application produces spatial density maps to ease the monitoring and decision making process by health professionals. We also include a discussion of how spatial density maps may be useful for health practitioners in such contexts.

**Conclusions:**

In this paper, we developed web-based client application and a set of geoprocessing web services to support specific health-spatial requirements. Spatial density maps of TB incidence were generated to help health professionals in analysis and decision-making tasks. The combined use of geographic information tools, map viewers, and geoprocessing services leads to interesting possibilities in handling health data in a spatial manner. In particular, the use of spatial density maps has been effective to identify the most affected areas and its spatial impact. This study is an attempt to demonstrate how web processing services together with web-based mapping capabilities suit the needs of health practitioners in epidemiological analysis scenarios.

## Background

According to the World Health Organization (WHO), the estimated global burden caused by Tuberculosis (TB) in 2009 comprises of 9.4 million of incident cases, 14 million of prevalent cases, 1.3 million deaths among HIV-negative people, and 0.38 million deaths among HIV-positive people [1]. Unfortunately, only 11% of MDR-TB cases (Multidrug-Resistant), i.e., 30475 out of 280000, were enrolled on treatment [2]. These negligent epidemiological situations are due in part to the fact that TB is a forgotten disease and its current long treatment has kept invariable since last 40 years [3].

Although most TB cases occur in developing countries, TB also strongly affects the inner cities of developed countries. For example, in Barcelona, the TB incidence rate in the inner city is 3 or 4 times greater than in the whole city [4]. This is due to the highest prevalence of TB risk factors in the poorest areas of large cities. In any of these cases, the inclusion of Geographic Information Systems (GIS) into epidemiological research can notably contribute to the control, analyses, screening/monitoring, intervention assessment, management and decision-making related to this old disease. GIS tools and related applications not only allow for the integration of complementary data and the introduction of the spatial dimension, but also to clearly support this dimension in processing medical statistics and managing TB threat situations.

To enhance rapid decision-making in such situations, it is of great importance to have all the appropriate and required information in place [3,5]. Beyond this, useful manners of presenting and visualizing such data sets are crucial to help health practitioners in their decision-making process. Examples of traditional presentation ways are graphics (plots, chart bar chart, etc.) tables, and even reports.

Nevertheless, location often plays a key role in health information, as in the case of epidemiological data [6]. On one hand, since this kind of information is geolocated, the best way to present it is by means of map-based tools, which allow users to study spatio-temporal distributions and evolutions of patients [7]. On the other hand, nowadays decision making processes involve several people in different places and requires information coming from distributed sources. Thus, the challenge is not only to present information on maps properly, but also to keep resulting information up to date.

With all these restrictions in hand, the intended application should be able to:

• visualize epidemiological data over up-to-date base cartography by using friendly, easy-to-use interfaces especially designed for non-experts in GIS.

• compute statistical analyses and processing over geocoded TB data.

• access to distributed information sources and resources.

As opposed to isolated and centralized solutions, the use of distributed processing capabilities and remote communications are essential components to share, visualize and interpret health data collaboratively. In this context, recent Web technologies and standards-based web services deployed on geospatial information infrastructures [8] are paving the way towards a new set of geospatial services for varied domain scenarios [9]. Distributed geoprocessing services are gaining attention as a way to access geoprocessing computations analyse capabilities in a web service environment [10]. Thus, geo-processing computations are software processes that simulate and analyze some kind of real-world phenomena in disparate domain applications [11].

The establishment of geospatial information infrastructures for sharing and delivery of data and services across environmental and health domains is an emerging trend [12-15]. The intrinsic geographic component in epidemiological data has led to several projects and applications that use geospatial web services to explore, share, and visualize such data sets on the spatial dimension [16-19]. Web as an integration platform is having a great success, for instance, Kamel Boulos et al. reported on the application of recent services APIs and modern technologies to build interactive health mashups [20,21]. Nevertheless, none of the previous works has focused on the Representational State Transfer (REST) architectural style. The question we pose here is whether REST may be applied to the development of applications that ease the access, integration, processing, and visualization of epidemiological data.

In short, REST [22] is a set of design principles and constraints that guide the design of resource-oriented applications in distributed systems. The application of REST principles yields RESTful services and applications composed of resources [23]. In this sort of applications, clients interact directly with the exposed resources through a uniform interface, materialized basically through the combination of HTTP (Hypertext Transfer Protocol), URI (Uniform Resource Identifier) and standard formats such as HTML (Hypertext Markup Language) and XML (Extensible Markup Language).

In the literature, several successful resource-oriented applications and RESTful services are emerging in diverse domains such as bioinformatics [24], genome analysis [25,26] and biodiversity [27,28]. However, to the best of our knowledge, RESTful approaches have been scarcely considered yet in health domain. Furthermore, the aim of this paper is the development of a web-based application and RESTful geospatial services that compute health data to produce spatial density maps over TB data in Barcelona during 2009.

## Methods

In this section the design of the system architecture and the main constituent components and services are presented, as well as the description of data sets, existing tools, and frameworks used.

### Conceptual architecture and essential components and services

**Figure 1:**
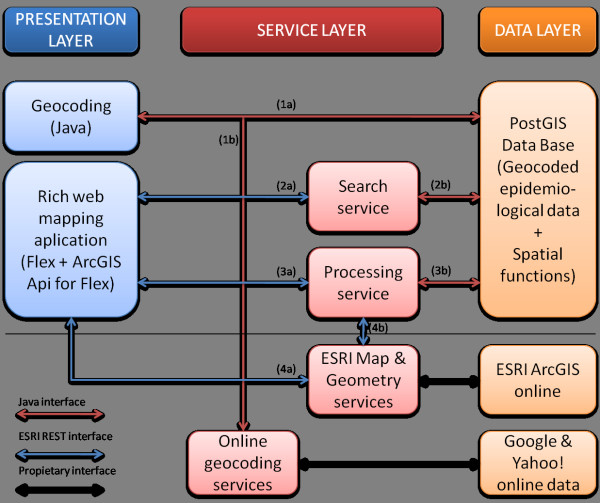
**Implemented architecture and essential components and services**. The architecture consists of three main layers: presentation, service and data layers. The components above the horizontal line has been implemented in our application, while the services and data bases bellow are third-party components (re-)used on our application.

To implement distributed, scalable web application to support mapping and geospatial processing over TB cases, we rely on a resource-oriented architecture and RESTful services, as introduced earlier. Figure 1 illustrates the proposed conceptual architecture that integrates all of the components and services. It is composed of three layers - a presentation layer, a service layer, and a data layer- and provides the guidelines to explain and describe the methodology used in the following subsections.

The presentation layer contains the developed client applications that interact with the set of RESTful services in the service layer. Two different but complementary applications were developed: a desktop-based geocoding application and a web-based mapping application.

Geocoding is essential for data preparation, and therefore a geocoding application has been developed, aimed to guide health professionals without geographic knowledge. The web-based mapping application is meant for visualizing and interpreting, spatially, health data. It allows users to interact with a potential range of geoprocessing services to perform diverse analysis tasks.

The service layer implements the set of RESTful services to support search and processing capabilities over epidemiological and geospatial data repositories. Such repositories form the data layer. TB data used in our experiment is stored in a local database while related base maps and cartography come from remote repositories.

### Data

The Barcelona TB Program is divided into four sub-programmes: case detection, patient control and treatment follow-up, prevention, and social support. In the case of the detection sub-programme, active and passive epidemiological surveillance is carried out. Passive epidemiological surveillance is based on physicians' notifications, while active surveillance is based on the evaluation of microbiological results, hospital discharge records and registers for TB cases, drug users and mortality [4]. By combining active and passive surveillance methods, Barcelona TB staff maintains a patient record which includes, among other properties, the place of residence. In particular, for the study described in this paper, we utilized TB data of all patients registered in Barcelona during 2009 (almost 400).

To support the spatial visualization of such TB data, we make use of base maps available online. For our study, base map and reference layers were obtained from ArcGIS Online servers [29]. ArcGIS Online layers are requested by our web-based mapping application to be mashed up with the TB data.

### Database loading

As the source database with TB data did not support explicitly geographic features, we migrated all of the data for our experiment to a spatial database with native support for geographical types and spatial functions. After evaluating several open source alternatives, the combination of PostgreSQL [30] and the PostGIS spatial extension [31] was the database technology of choice due to its reliability and the extensive documentation available. PostgreSQL is a relational database, while the extension PostGIS is uniquely dedicated to implement geospatial characteristics. Both, PostgreSQL 9.0.1 and PostGIS 1.5.2, offer full integration with Java that is the programming language used in developing the geoprocessing web services, as discussed later on.

For compatibility, we decided to replicate the source database schema into the target database. The only exception was a new column, called *geolocation*, of type geometry point to store the position (geographical coordinates) for each TB case. In addition, various views were created to serve geocoded data. A database view consists of a stored query which may represent a subset of the data in one table or an aggregation of various tables. In our case, views are used to join multiple tables and hide the complexity of the query from the developer's perspective. For instance, one of the views extracts the longitude and latitude of the column *geolocation*, by combining some PostGIS built-in stored procedures (*st_x *and *st_y*).

### Geocoding of data

Once completed the loading of TB data into the spatial database, the next step consisted of turning such data into geographical-aware data in order to study the spatial density and distribution of TB cases. This process is known as geocoding and its importance for epidemiological research studies is well documented [32,33].

The process of geocoding offers two complementary methods: geocoding and reverse-geocoding. The former deals with the extraction of the current position in terms of coordinate pairs from textual location descriptions, such as postal addresses and place names. This method is commonly used throughout health-related research, because health data often only contain low accurate position descriptions like district names and administrative units.

The latter method, the reverse geocoding, is the opposite of the geocoding method. It consists of finding a concrete postal address, place name or any textual location description for a given pair of coordinates. We used the geocoding method because patient records in our study include location descriptions.

One of the main problems of geocoding is the spatial resolution used and the error in positional accuracy of the resulting geocoded data [34]. The literature regarding the positional accuracy of the geocoding process [35,36], and the effects of varying the geocoding services used is extensive [37]. Errors in positional accuracy for geocoded addresses are unavoidable and depend on many factors, such as the quality of initial data and the inherent accuracy of commercial geocoding sources used. Bonner et al. [38] have found that geocoding of addresses, above all in populated cities, is often very accurate regardless of the geocoding source used. In addition, Goldberg et al. [36] proposed a manual correction of the geocoding driven by a web-based interactive approach to improve the quality of geocoded data.

Given the characteristics of our study, focused on a delimited geographical area (metropolitan area of Barcelona) and the relative low number (around 400 for 2009) of TB cases, we decided to use a hybrid approach based on [38] and [36]. It combines existing geocoding services available online and post manual correction of the resulting geocoded data. We have developed a desktop-based Java application that helps users in geocoding and managing large amounts of geocoded data in two steps:

First, it automatically performs the geocoding of home residence addresses using the Yahoo! Place Finder service [39]. We analyzed and conducted some experiments on four commercial geocoding services [39-42]. The selection of Yahoo! Place Finder service was based on accuracy (although Google Maps offers similar results) and, especially, due to the variety of supported response formats.

Second, users can check visually the resulting coordinates on a map (Yahoo Maps or Google Maps). In case of ambiguity or uncertainty, users themselves may modify manually the pair of coordinates, either in latitude/longitude or in UTM (Universal Transverse Mercator) coordinate system, by simply selecting the exact point in the map.

**Figure 2:**
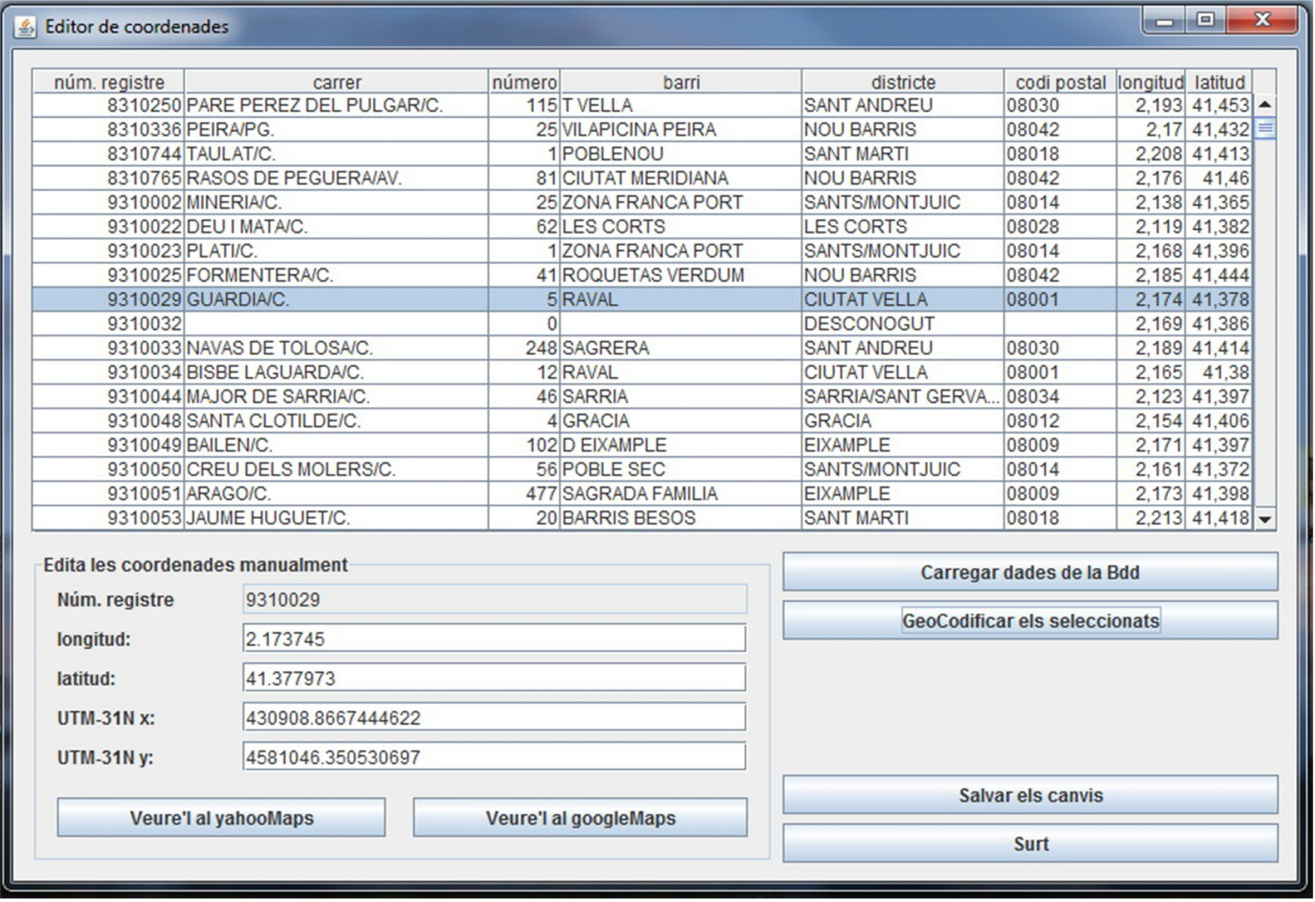
**User interface of the geocoding application**. This Java-based application allows users to load database records (Tuberculosis cases), perform automatic geocodification through remote geocoding services, assess visually on the map the results, and save the validated geocoded data to the database.

The geocoding application guides users through the geocoding process as follows: (i) the user selects the data records from the database that are going to be geocoded, step (1a) in Figure 1; (ii) then, in step (1b) in Figure 1, the desktop-based geocoding application constructs the input parameters, calls the Yahoo! Place Finder service, and reads the response (See Figure 2). The input parameter is the patient's home address composed of type of street, street name and postal code. In the following step (iii), the user inspects visually the geocoded data on the maps (See buttons labelled with "*Veure'ls al yahooMap" *(View on Yahoo Map) and "*Veure'ls al googleMap*" (View on Google Map) in Figure 2). Finally, (iv), in step (1a) in Figure 1, geocoded values are stored into the database by selecting the button labelled as "*Salvar els canvis*" (Save) in Figure 2.

It is worth mentioning two aspects in our experiment. The first aspect is concerned with protecting patient's privacy [43]. We took some measures to deal with this issue. First of all, personal information (name, phone number, etc.) was completely omitted from the TB records used in the application; second, we performed some significant changes to mask data used in our experiment [44]: (i) the floor number of the patient record was omitted; and (ii), the final street number was masked by adding +1 or -1 to the original street number on a random basis. Thus, for confidentiality reasons, we assumed certain error in accuracy in the resulting geocoded data. The positional error in the geocoding process depends largely on the completeness of the source data (street name, street number, etc.). As most of TB records contained full address (except floor numbers, as earlier commented), the accuracy of the geocoding service was very precise, since street name and masked street numbers were provided as input parameters to the geocoding service. In a few cases, though, some parts of home addresses (e.g. street number) were missing and the final geographic location was represented by the centre of the street in question. It is important to note that the errors introduced to mask confidential data do not affect the results of the experiment, because, as will be shown later on, the exact location is not so important in generating spatial density maps.

The second aspect refers to multilingual issues. We had to replace some special characters used in Spanish and Catalan (e.g. "ñ", "ç" which are not present in English) with the equivalent HTML characters ("ñ" and "ç" respectively). Multilingual aspects involve a pre-processing step in converting the textual home addresses into compatible HTML-encoded strings.

### Development of the web-based client application

Having georeferenced TB data, the next step is the development of the web mapping application to support decision-making processes. Rich Internet Applications (RIA) are characterised by a high level of dynamisms and interactiveness, which fits nicely with the requirements of web mapping applications, like the one presented in this paper. Our RIA technology of choice has been Flex [45] and ArcGIS API for Flex [46].

On one hand, Flex eases the creation of expressive, interactive user interfaces using a great variety of ready-to-use widgets and components, which reduces greatly development time and resource efforts necessary for quick prototyping. On the other hand, ArcGIS API for Flex integrates smoothly in Flex-based applications and provides dedicated methods and functions to access and interact with remote spatial services, not only ESRI servers but also other kinds of map servers like OGC services (e.g. WMS).

### Development of RESTful geoprocessing services

All geoprocessing services have been implemented based on the ESRI GeoServices REST specification [47]. In REST, client applications issue HTTP requests through the common HTTP methods (e.g., GET, POST) over the set of resources exposed in a server. Such resources are identified by means of HTTP URIs and can be of any nature such as spatial search, map services and geoprocessing services. The HTTP protocol is used in RESTful services as an application protocol to interact with any type of resource in the server side. This approach provides several benefits for application development [23]: simplicity, through exploiting common web standards such as HTTP and URI; scalability, since the addition of new resources does not break with existing client applications due to the uniformity of the access interface; and independency, since servers and clients are loosely-coupled and can evolve independently without limiting interoperability.

In order to support the requirements of our study, we have developed two types of RESTful services: (i) a search service, which allows clients to search for specific TB cases according to thematic (age, etc), temporal (month, year) and spatial (a certain geographical area) criteria; and (ii) a processing service, which computes spatial density maps and counting, i.e., the sum of cases in a given geographical area delimited by the user through the web-based application. Additionally, the proposed processing service may rely on other remote services that provide geospatial computations so as to harness external processing resources as being part of a complex analysis task.

Nevertheless, we still needed a data model for the proposed RESTful services capable of emulating the request-response communication between ESRI clients and RESTful services. In doing so, we benefited from being compliant with the GeoService REST specification in two aspects: first, any of the ESRI client APIs (JavaScript, Flex, and Silverlight) may be used to develop the front-end without modifying the set of RESTful services; second, the amount of code necessary on the client application was drastically reduced since such APIs already support the RESTful communication interface [47].

#### Common data model

The proposed search and processing services reflect the behaviour of Map Services [47] that basically expose Layer and Table resources. In our case, we restricted to Table resources because our TB data is stored in a spatial relational database. A Table resource supports the Query operation [47], which provides as a result a set of feature objects with geometries and attributes, i.e., the spatial and non-spatial data attached to an individual TB case.

**Figure 3:**
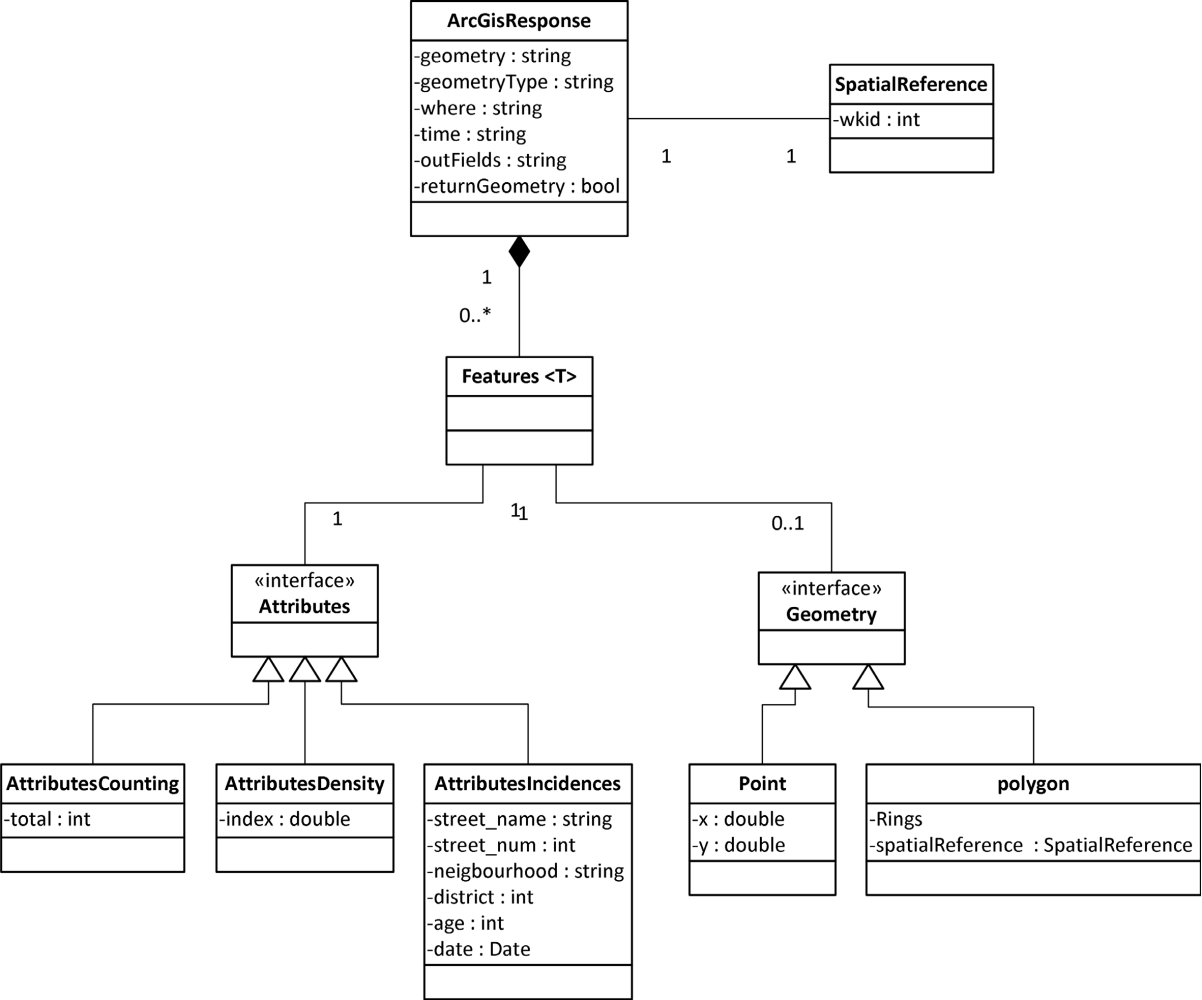
**Class diagram of data model of the restful services**. Implemented data model to support responses compliant with the ESRI Geoservices REST specification.

Figure 3 shows the developed data model as a set of Java classes that respect the ESRI naming conventions and types for the set of features objects and properties. The structure of classes of the data model facilitates automatic serialization of the responses in JSON (JavaScript Object Notation) [48], a common standard notation for structured data which is less verbose than XML, as the preferred response format [47].

For the request, the most relevant parameters required by the specification are mapped to the data model in Figure 3 as follows:

• InSR parameter, which identifies the coordinate reference system, is mapped to the *wkid (well-known id) *property of the *SpatialReference *class.

• The following parameters -geometry, geometryType, where, time, outFields, and returnGeometry- are mapped together as properties in the *ArcGisResponse *class. The *geometry *and *geometryType *properties define a spatial filter, for example a polygon or point defined by the user. The *where *and *time *properties contain thematic and temporal filters respectively. The *outFields *property lists the attributes returned in the response, such as street name, age and district. Finally, the *returnGeometry *informs whether the response contains a geometry associated.

For the response, the object *Features *is the only required object, which is a list of objects with geometries and attributes. In our study, the list corresponds to the requested TB cases. Each object in the *Features *list contains: a *Geometry *object with two coordinates, *x *and *y*; an *Attributes *object that contains all of the properties associated to a given geometry. In particular, these properties belong to the patient's medical record such as age, neighbourhood, district and street name.

The search and processing services operate in the same manner. Both initialize an instance of the *ArcGisResponse *class with the requested parameters and populate the remaining classes with the results in function of the query requested, either search or processing functionalities, as explained in the following.

#### Search service

The search service performs queries over the epidemiological database (TB cases) based on thematic, temporal and spatial criteria specified by users. Thematic search parameters correspond to attributes of TB records such as age and genre. Temporal search parameter delimits a period of time in terms of starting and end dates. Spatial search parameter defines a geographic area of interest.

The proposed search service emulates the ESRI's Query operation on Table resources. It also supports spatial requests and geometry information embedded in the response, as spatial queries are not supported against Table resources [47].

Once the search parameters are collected (step (2a) in Figure 1), we build a spatial query (view) by combining various PostGIS built-in stored procedures to recreate a virtual polygon according to the requested area, and filter the TB records that are within that virtual polygon. In the step (2b) in Figure 1 the following built-in stored procedures were used:

• *ST_GeomFromText: *it transforms the requested bounding box, i.e., a string of four coordinates into PostGIS geometries of type Point.

• *ST_MakePolygon: *it creates a virtual polygon from the previous points.

• *ST_SetSRID: *it assigns the coordinate system of the virtual polygon according to the requested coordinate reference system.

• *ST_Within: *it performs a spatial operation to find the TB incidence records that fall within the virtual polygon generated. For performance, we created a GiST (Generalized Search Tree) index on the geometry column of the table of TB cases.

**Figure 4:**
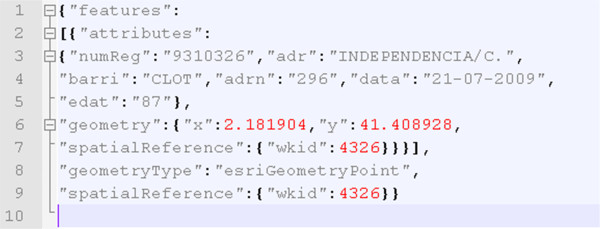
**A JSON response generated from the query restful service**. These responses are typically much more extensive. The excerpt above shows one JSON-encoded incidence that contains properties and spatial information to be processed in the client side.

The values of the resulting record set are mapped to the *AttributesIncidences *class and the Point class (see Figure 3), which are thus serialized in JSON format. Figure 4 shows an example of the JSON response of the query service forwarded to the client application.

#### Processing service

The processing service performs a couple of tasks. On the one hand, it computes a spatial density map and, on the other hand, it also counts the occurrences of TB cases in a given geographical area. Both tasks consider the search criteria mentioned earlier, i.e., the spatial density map and counting processes are generated according to the thematic, temporal and spatial criteria specified by users.

As in the case of the search service, the processing service is built upon the ESRI's Query operation on Table resources. In function of the *returnGeometry *search parameter, the processing service performs one or other function. When *returnGeometry *is set to false, the counting process is carried out since no geometry is returned, only a number as the sum of TB cases. Otherwise, the spatial density map process is executed.

For the spatial density map calculation, the selected geographic area (bounding box) is split in a square grid (tessellation) and each cell contains a relative density index. After some tests, we decided for constant cell size to achieve a right balance between quality of the results obtains and performance. The density index is calculated as follows: for each cell the geometric centre (centroid) is computed and then a buffer is calculated around this point to count the TB cases within the zone of influence. The relative index is a normalized value with respect to the cell area.

Once the search parameters are collected, step (3a) in Figure 1, we build again a spatial query by combining various PostGIS built-in stored procedures to generate the square grid with a density index. In the step (3b) in Figure 1, apart from the *ST_GeomFromText*, *ST_SetSRID*, and *ST_Width*, we utilized two additional built-in stored procedures for the spatial density map process:

• *ST_Centroid*: it computes the geometric point of a given geometry, a square polygon in our case.

• *ST_Buffer*: it computes a spatial buffer around a given point (centroid).

For the counting process, the calculation is similar to the search service but differs in the response. Rather than returning a list of TB cases with attributes and geometries, the response only returns the sum of TB cases by using the *count *function, a standard database function.

Results are returned according to the data model depicted in Figure 3. In the spatial density process the class *AttributesDensity *is used, whereas the class *AttributesCounting *in the counting process.

#### Remote geometry services

The geoprocessing services described earlier use spatial and geometry operations built in spatial databases. Opposed to this database-centric solution, an alternative approach is to delegate some spatial processing to remote services. Some examples are distributed geoprocessing web services [9] and the ESRI Geometry Service [49]. The latter provides among others buffer and lengths operations (calculation of geodesic distance among geometries). In particular, these remote operations are called directly from the client applications, step (4a) in Figure 1, using the ArcGIS API for Flex [46], since the Geometry Service is one of the public ESRI REST Geoservices. Nevertheless, such operations may be also used from the processing services, step (4b) in Figure 1, where appropriated.

## Results and Discussion

This section describes the capabilities of the proposed application in terms of the services developed, as well as the implications they have in epidemiological analysis tasks.

When the user opens the web-based mapping application, all of the TB cases are retrieved and displayed on the map as red circles over base cartography. Each circle is queryable by clicking on it in order to inspect the attributes associated to the selected TB case. As the area of study is centred exclusively on the city of Barcelona and the number of cases is relatively moderated, it is not a critical restriction to show all the geocoded epidemiological data on the map without considering filtering parameters. Nevertheless, it is worth noting some scalability aspects when the number of TB cases increase up considerably. On the one hand, the search service performs queries in the server side, i.e., using built-in database capabilities. In addition, GIST indexes help in speeding up spatial-based queries, within the database. In this sense, scalability concerned with an increasing number of cases stored is delegated to the database system. On the other hand, visualization of a great amount of TB cases is critical due to network factors such as network latency and the size of exchanged messages between services and clients [50]. The use of cache mechanisms in the client and compression of exchanged messages would reduce response times and unneeded request-response interactions.

**Figure 5:**
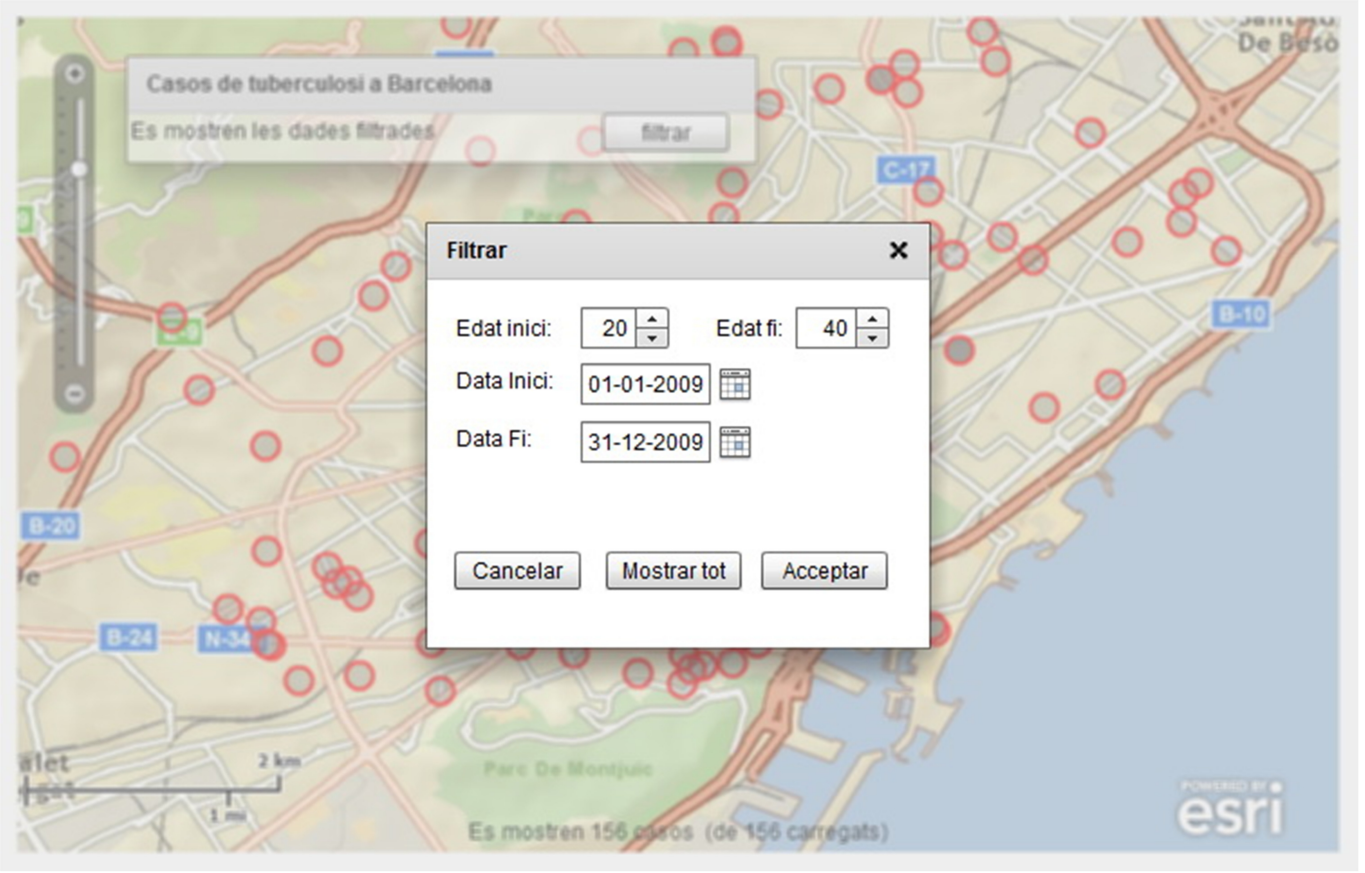
**User interface of the web-mapping client application with the filtering options**. Thematic (age) and temporal search criteria are introduced by the user. Spatial search criterion is automatically set according to the current bounding box, i.e., the visible geographic area in the client application.

Once the initial configuration takes place, the user interacts with the application by selecting the filtering option on the contextual menu. This opens a new window where a user can enter the thematic and temporal search criteria (See Figure 5). Selecting the "*Acceptar*" (Accept) button implies to perform a call to the search service described in the earlier section. JSON-encoded results are rendered by the client application as a new layer in the map viewer. As before, each TB case in the resulting set is represented as a queryable red circle.

**Figure 6:**
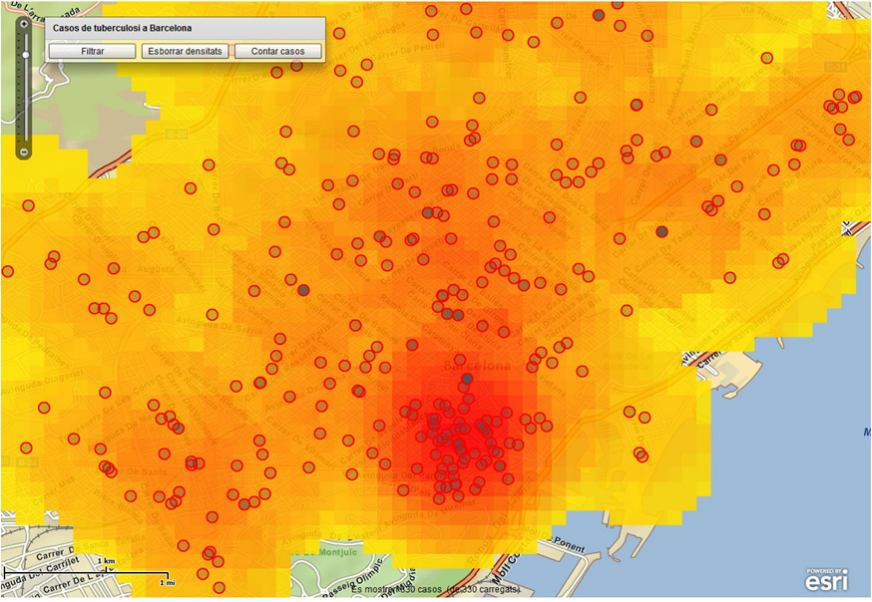
**Example of spatial density map**. This map represents the spatial density map calculated by the processing service.

The objective of the spatial density maps is to detect "risk" areas with high concentrations of TB cases, i.e., the identification of emerging urban clusters that deserve a special, dedicated analysis [51]. Spatial density maps are easily generated through our web-based client application. The user generates it by simply selecting the "*densitats*" (Calculate density maps) option on the contextual menu. This triggers the corresponding processing service as described in the previous section. Again, the resulting map is rendered by the client application as a new layer on the map viewer (see Figure 6).

It is worthwhile commenting that the generation of the spatial density maps is also driven by thematic, geographic and temporal search criteria. These maps can then be easily customized to specific needs, such as density maps restricted to a certain geographical area, range of age, or a given period of time. Indeed, from the perspective of health professionals, this functionality becomes a powerful analytical tool to enhance decision making. The density maps are very spectacular and shed some light on the understanding of the TB problem. Figure 6 shows how concentration of TB cases is higher in the inner city that corresponds to the *Ciutat Vella *district. In particular, two quarters (*Raval Nord *and *Raval Sud*) of the *Ciutat Vella *district have the highest TB incidence rate of 75.8 and 114 per 100000 inhabitants respectively (see Table 7 in [52]). This is not surprising as the population in this area has a high prevalence of TB risk factors (alcohol abuse, injecting drug use, homeless, low socio-economic level, overcrowding, etc.). However, the latter statement should be understood as the result of a follow-up analysis with socio-economic statistics of the area, rather than a result derived directly from the spatial density maps.

**Figure 7:**
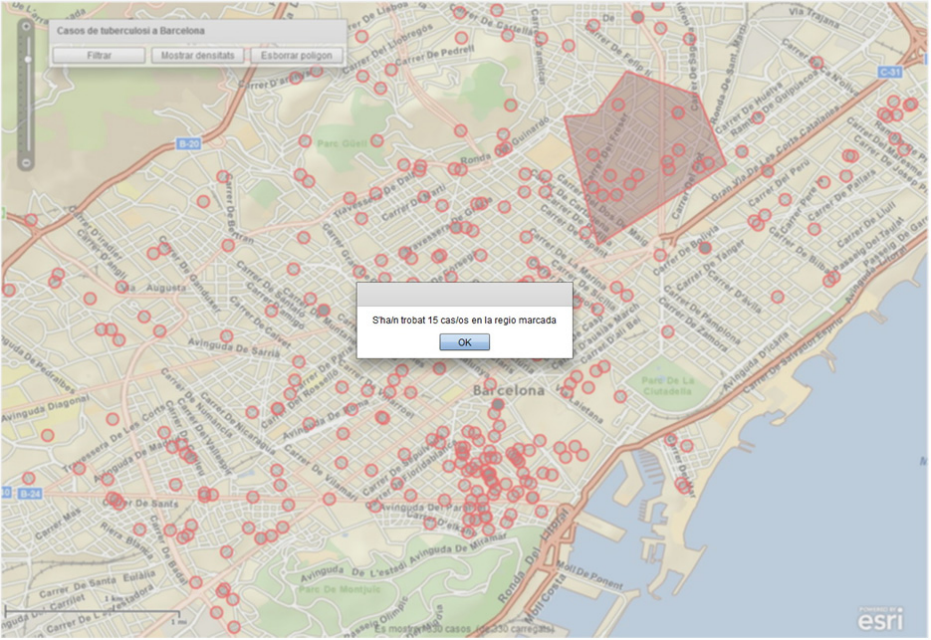
**Example of spatial user selection and counting result**. This map shows the spatial selection delimited by the user and the counting result calculated by the processing service.

Apart from the generation of spatial density maps, the second functionality exposed by the processing service aims at counting the number of cases in a user-delimited area. This area is not limited to a rectangular zone or bounding box, but can be also an irregular polygon. In particular, a user selects the "*contar casos*" (Count TB cases) option on the contextual menu which automatically enables the drawing tool to let the user to delimit the desired area on the map. This action in the client side automatically invokes the counting processing service to calculate the occurrences of TB cases in such an area (see Figure 7). Again, the search criteria (thematic, temporal, and geographic) are considered in the counting processing service. As the amount of epidemiological data increases, the counting function gains in importance because it can be applied for several needs, like counting cases in a given area in a period of ten years.

**Figure 8:**
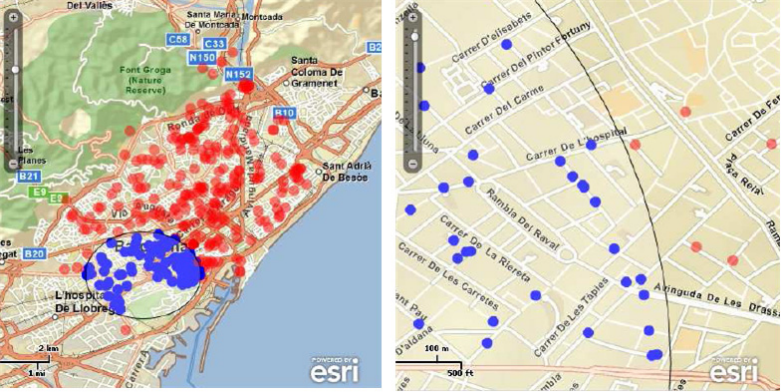
**Example of buffer result**. The left map shows a zone of influence around a central point selected by the user and calculated by remote services, while the right map shows a zoom in on the boundary of the zone of influence.

Finally, the ability to integrate remote geoprocessing services in the proposed client application and services (see Figure 1) is illustrated by a buffer operation. Users can calculate the zone of influence (buffer) by simply selecting an individual incidence on the map, and choosing a distance around the central point. This action triggers an asynchronous call to the buffer operation of the remote ESRI Geometry service, as described in earlier section. Figure 8 exemplifies the creation of a buffer to 2000 meters from the *Plaça Espanya *in Barcelona. The buffer or zone of influence is represented by a transparent white circle with black border. TB cases within the zone of influence are displayed as blue circles, so that these can be easily distinguished from those cases out of the zone. It is important to note that the buffer operation is aimed here to illustrate the integration of remote geoprocessing services rather than proving a utility to calculate a sophisticated exposure area for a given TB cases [53].

## Conclusions

In this paper, we developed both a rich, web-based client application and geoprocessing web services to support specific health-spatial requirements, as spatial density maps of TB incidence, and help health professionals in analysis and decision-making tasks.

On the one hand, the combination of GIS and geoprocessing in TB scenarios leads to interesting possibilities, and allows health researchers to apply new methods in handling health data in a spatial manner. As a proof-of-concept experiment, the proposed web application may be of great importance for monitoring the spatial and temporal dispersion of TB cases in Barcelona, since traceable information provides elements necessary to explain problems of a particular area of interest. For instance the use of spatial density may be an effective mechanism to identify the most affected areas, it may also serve as a basis to forecast TB immediate impact (together with complementary information), and finally it may be used to carry out post-evaluation programs by analyzing annual changes between spatial density maps over different years. The combination of these benefits may result in direct, specific intersectional actions, and thus provide for better analysis, control and decision-making.

On the other hand, from the technical perspective, the combination of REST-based services, open source software (PostGIS and JAVA), and free proprietary software (Flex and ArcGIS API for Flex), provides a suitable framework to build rich, scalable web applications. Some of the services proposed throughout this paper are functionally similar to some ESRI services, though, the restriction imposed in terms of open source software and limited budget impede the acquisition of licenses of such commercial products. However, by simulating the behaviour of the ESRI protocol specification with open source components, we take for granted the benefit of free (both commercial and open source) technology already in place to development rich web applications.

Health research is heading towards a multidisciplinary field in order to respond to the new scenarios on public health and their consequences for society. Such new contexts can only be addressed from a collaborative perspective where socio-economic, health, geospatial, and environmental data are integrated together. Our future work will be focused on the addition of specific geoprocessing web services so as to include socio-economic dimensions in the TB analysis tasks coming from open government data repositories such as OpenData BCN [54].

## List of abbreviations

GiST: Generalized Search Tree; HIV: Human Immunodeficiency Virus; HTML: Hypertext Markup Language; HTTP: Hypertext Transfer Protocol; JSON: JavaScript Object Notation; OGC: Open Geospatial Consortium; REST: Representational State Transfer; TB: Tuberculosis; MDR-TB: Multidrug-Resistant Tuberculosis: UTM: Universal Transverse Mercator; URI: Uniform Resource Identifier; WHO: World Health Organization; WMS: Web Map Service; XML: Extensible Markup Language.

## Competing interests

The authors declare that they have no competing interests.

## Authors' contributions

PD developed the geocoding tool, developed the geoprocessing services, developed the web-based application, and wrote the manuscript. CG conceived of and designed the study, coordinated its execution, and wrote the manuscript. AP participated in the design of the study, coordinated its execution, and provided comments and suggestions on the manuscript. MC, AO, and JC provided the epidemiological data of the study, and provided comments and suggestions on the manuscript. All authors read and approved the final manuscript.
